# Medical Literature of Philadelphia

**Published:** 1860-05

**Authors:** 


### NORTH AMERICAN

### MEDICO-CHIRURGICAL REVIEW.

MAY, 1860.

### Analytical and Critical Reviews.

ART. I.—*Discourse on Comparative Anatomy.* By JOHN MORGAN, M.D.

*Medical Inquiries and Observations.* 4 vols. By BENJAMIN RUSH, M.D.

*Medical Inquiries and Observations on the Diseases of the Mind.* By BENJAMIN RUSH, M.D.

*Medical and Physical Memoirs.* 2 vols. By CHAS. CALDWELL, M.D.

*Observations on the Causes and Cure of Remitting or Bilious Fever, etc.* By WILLIAM CURRIE, M.D.

*Memoir on the Analysis of the Black Vomit.* By ISAAC CATHRALL, M.D.

*A Treatise on Malignant Fever, etc.* By STUBBINS FFIRTH, M.D.

*A System of Anatomy for the Use of Students.* By CASPAR WISTAR, M.D.

*Elements of Botany, etc.* By BENJAMIN SMITH BARTON, M.D.

*Elements of Surgery for the Use of Students.* By JOHN SYNG DORSEY, M.D.

*The American Dispensatory.* By JOHN REDMAN COXE, M.D.

*Elements of Therapeutics and Materia Medica.* By NATHANIEL CHAPMAN, M.D.

*The Institutes and Practice of Surgery.* By WILLIAM GIBSON, M.D.

*Dispensatory of the United States.* By GEORGE B. WOOD, M.D., and FRANKLIN BACHE, M.D.

*Vegetable Materia Medica of the United States; or, Medical Botany.* By WM. P. C. BARTON, M.D.

*Diseases of Females.* By WM. P. DEWEES, M.D.

*A Compendious System of Midwifery.* By WM. P. DEWEES, M.D.
*Obstetrics: the Science and the Art of.* By CHAS. D. MEIGS, M.D.
*Females and their Diseases.* By CHAS D. MEIGS, M.D.
*A Treatise on the Practice of Surgery.* By HENRY H. SMITH, M.D.
*Therapeutics and Pharmacology.* By GEO. B. WOOD, M.D.
*Dictionary of Medical Science.* By ROBLEY DUNGLISON, M.D.
*Medical and Physical Researches.* By R. HARLAN, M.D.
*Therapeutics and Materia Medica.* By ROBLEY DUNGLISON, M.D.
*A Treatise on General and Special Anatomy.* By WM. E. HORNER, M.D.
*Operative Surgery.* By JOSEPH PANCOAST, M.D.
*Illustrated Anatomy.* By S. G. MORTON, M.D.
*Materia Medica and Therapeutics.* By THOS. D. MITCHELL, M.D.
*Practice of Medicine.* By JOHN EBERLE, M.D.
*A Treatise on the Practice of Medicine.* By GEO. B. WOOD, M.D.
*A Treatise on Baths, etc.* By JOHN BELL, M.D.
*A System of Surgery.* By S. D. GROSS, M.D.
*Manual of Diseases of the Eye.* By S. LITTELL, M.D.
*Medical Botany.* By JOSEPH CARSON, M.D.
*A Flora and Fauna within Living Animals.* By JOSEPH LEIDY, M.D.
*The Writings of Hippocrates and Galen epitomized.* By JOHN REDMAN COXE, M.D.
*Yellow Fever, considered in its Historical, Pathological, Etiological, and Therapeutical Relations.* By R. LA ROCHE, M.D.
*A Practical Treatise on the Diseases of Children.* By D. F. CONDIE, M.D.
*A Practical Treatise on the Diseases of Children.* By J. F. MEIGS, M.D.

IN order that our readers may not be kept in suspense respecting our purpose in introducing to their notice the titles of sundry works separated by considerable periods of time, and treating of so many different topics, we may as well say at once, that it is our intention to discourse somewhat on the Medical Literature of Philadelphia. The subject follows naturally enough the "Medical Institutions of Philadelphia," an historical outline of which we gave in the last number of this journal.

The medical (and the observation will apply to the general) literature of Philadelphia is not remarkable for originality or depth of research in any one department. Its characteristics are sobriety of thought, honesty of purpose, and plain, and, for the most part, lucid digests of existing facts and opinions, together with commentaries which display habits of study and reading, if not profound learning. Seldom is it embellished by classical allusions or graces of style; but, on the other hand, it is not often disfigured by random hypotheses, strained analogies, and turgid forms of expression. The prose may be at times dull, but it creates no feigned nor doubtful issue; and, with few exceptions, it is not prose run wild or mounted on stilts. If it fails to secure animated applause, it is, at least, safe from ridicule.

Supposing our appreciation of it to be correct, we must not, in the medical literature of Philadelphia, look for the gushing fountain and rapid flow of invention and discovery, but ought rather to compare it to the extensive reservoirs at Fairmount, which supply by numerous conduits all parts of the city with pure and salubrious water for every purpose of personal and domestic as well as manufacturing and sanitary requirement. So are the contents of the vast medico-literary reservoir at Philadelphia distributed to nearly every part of the United States, to allay the thirst for knowledge and to meet many of the wants both of the youthful student and of the matured practitioner. The machinery for this purpose is twofold: first, by the oral instruction of the professors of the medical schools; and next, by the written and published lore, contributed in part by these same professors, and in part by their medical brethren who are not engaged in teaching, but whose aspirations are in this direction, or who find incentives to authorship in the more diffused appetite for reading created by the delivery of lectures and the making of text-books. Whatever may be the shortcomings of its medical schools, in not meeting all the varied requirements of medical instruction, we can hardly fail to see that the distinction Philadelphia has acquired in medical literature is in a great measure owing to the atmosphere created by these schools and the germination of the seeds sown in their lectures.

Wanting some genial influences of this nature, the general literature of the City of Penn continues to languish. Why is it that, on the one hand, the medical department of the University of Pennsylvania, without endowment or official encouragement of any kind, but, instead, heavily taxed by the payment of a large annual rent for the use of its college building, should have exerted such a benign and diffusive influence on medical literature; and, on the other hand, that the Faculty of Arts, free from any such taxation, and salaried by the Trustees, should have done so little for general literature and science, either in productions of its own or in scattering the seeds from abroad, passes our comprehension. For more than a century the University of Edinburgh has had its chair of moral philosophy filled by an uninterrupted succession of profound thinkers and eloquent teachers—Adam Ferguson, Dugald Stewart, Dr. Thomas Brown, John Wilson, and in a similar line, although in a different chair, Sir William Hamilton, all of them distinguished in more than one walk of literature or science, whether it were history, mathematics, criticism, poetry, æsthetics, or logic. Of the successive incumbents in the chair of moral philosophy in the University of Pennsylvania, one, after long deliberation, sent out a "Search after Truth in the Science of the Human Mind." His successors seem to await the result of the search, which they suppose is being made by somebody, but themselves not caring to agitate the questions to which reference to the work itself must give rise. While we admit the ability and painstaking of the Faculty of Arts of the University, we must regret that its modesty, so rare in these days of "Young America," prevents the results of its labors from reaching the public eye and ear, or from being measured by public opinion, except at commencement anniversaries, on which occasion a column of a newspaper contains a list of the names of those who have passed examination, and a notice of the addresses of sundry young masters, all of whom are supposed to have completed their studies at an age when the aspirants for distinction in scholarship and literature feel that theirs have only properly begun. Who, at a distance, hear of the existence of the University of Pennsylvania as they do of that of Harvard or of the college at Princeton? How many, even of the citizens of Philadelphia, are aware of their having such an institution in the midst of them? And of those cognizant of the fact, how small is the number that care for it, or can, without a violent strain on their civic pride, connect its name with, we will not say originality of view and discovery, but with out-door progress even, in literature, science, or the fine and liberal arts! We have sometimes heard of the odd way in which a trustee of the University exhibits his opinion of its educational competency and his kindly desire to promote its success. It is neither more nor less than sending his son to a college down East, from which the youthful graduate returns, less indeed of a Philadelphian, but not always more of a scholar. "Call you that backing your friends?"

Whence this apathy, this evident want of concentrated efforts for literary advancement and of congenial feeling for an intellectual worker, whether he be poet or political economist, mathematician or moralist? The calm surface of Philadelphia literature is in no danger of being ruffled by underlying enthusiasm, and if there should, by any chance, be a gush of this latter sentiment, it is looked upon as a prodigy, or rather a portent, which drives the people from their propriety; and even they who for awhile indulged in it breathe more freely when it has subsided. We may advert, in illustration of our meaning, to the excitement caused by Dr. Kane's narrative of his Arctic expeditions and adventures. Both the ádventures and their history broke up for a season, in quite a startling manner, the monotony of Philadelphia public life and literature. This slowness of appreciation of whatever goes beyond the line of conventional agreement is caused much more by timidity and fear of change than by envy, to which it has been sometimes, but erroneously, attributed. Owing to this infirmity in the Philadelphia mind, there is a want of heartiness of co-operation among its citizens in general, and among the several classes of these, for the advancement of any new measure, whether it be commercial, financial, or literary. Voluntary associations, numerous and well intended as they are, fail to make up for this deficiency. They are the expression of a desire on the part of well-meaning associates to meet obvious wants and deficiencies, and so far call for sympathy. The very name and programme of the objects aimed at by a society are suggestive of kindred if not imitative and rival efforts in other directions. Even the failure to accomplish its original purposes incites to the trial of a different method to obtain the desired results. In this sense, to the American Philosophical Society may be attributed the origin of the Academy of Natural Sciences and the Historical Society, and to the University of Pennsylvania the High School and the Polytechnic School. Other secessions, such as that from the Academy of Fine Arts, have not been equally successful, although the necessity still exists for a good school of art properly endowed and put in working order. Let us not fail, however, to speak in becoming terms of praise of the exertions of those who founded and sustained the institution just named. If the rectangular streets and the drab coats of the City of Penn and his companions and their descendants have proved to be inauspicious to poetry, they have not interfered with the waving lines and varied hues spread by the painter on his canvas, when he would portray the human face divine, or exhibit scenes of rural beauty. In each of these departments, or of portrait and landscape painting, Philadelphia art has competed well with rival exhibitions from other quarters, while biding her time to contest the palm for the more studied compositions and groupings, and the bolder contrasts and greater depth of color, in historic painting.

It must be acknowledged, before closing these incidental remarks, that although Philadelphia is often backward in the suggestion and slow in carrying out improvements of various kinds, yet when the thing is done, it is well done. Resting on a knowledge of this kind, we may reasonably hope that when her people do apply themselves earnestly to literary productions of a high order, they will go beyond those of their neighbors who are now in advance of them.

The medical literature of Philadelphia can scarcely be said to have begun or exhibited any noticeable features until after the war of Independence. The slight contributions furnished by Dr. Thomas Cadwallader, on the "Iliac Passion," in 1740; by Dr John Kearsly, Jr., on "Angina Maligna," in the Gentleman's Magazine for 1769; and papers, the titles of which are unknown to us, by Dr. Thomas Bond, in a London periodical; and the discourse on Comparative Anatomy, by Dr. John Morgan, may serve to gratify bibliographical curiosity without making any substantial addition to our present subject. It is somewhat surprising, indeed, that there should be no history of the yellow fever of 1762, save in a note kept by Dr. Rush, then a youth only seventeen years of age, and in a letter addressed by Dr. Redman, an eminent practitioner, to the College of Physicians in 1793.

With the writings of Dr. Benjamin Rush begins, properly, the medical literature of Philadelphia, and may we not say also of North America? They have been published in four volumes, under the title of "Medical Inquiries and Observations." In another separate volume, but with the same general title, is the treatise on "The Diseases of the Mind." They embrace a great variety of topics—physiological and psychological, hygienic, therapeutical, and ethical—and evince a wide range of inquiry and acuteness of observation, blended with ingenious and sometimes fanciful speculations. All of them wear an air of freshness, are eminently suggestive, and are pervaded by such a high moral tone and benevolent spirit as to make the reader in love with their author, even when at times he may demur to the soundness of his argument. Seldom is an attempt made to carry out a proposition to its ultimate consequences by a systematic process of rigid induction. More generally the thing affirmed is sustained by the citation of analogous facts which have come under the observation of the author, or are furnished by his reading. Without any parade of learning or effort at intellectual fence, he handles his materials with the ease of a man who has thought long and earnestly on his subject, and is desirous that others should feel the same interest in it that he does himself. We may be disappointed in not finding profound research, but, in return, we are sure to be gratified with the frequent exhibition of broad and comprehensive views, suggestions which have, in other hands, become discoveries, and predictions, or the effusions of sanguine hope, which, in their subsequent realization, seem to border on prophecy.

We shall first notice the discourses of a more general nature. Of these the one which takes precedence on the score of date is "An Inquiry into the Natural History of Medicine among the Indians of North America, and a Comparative View of their Diseases and Remedies with those of Civilized Nations." This was read before the American Philosophical Society on the 4th of February, 1774, as an anniversary oration, and as a substitute for one that was to be delivered by Mr. Charles Thompson, soon to be known and celebrated as secretary to the first Continental Congress, which met in the month of September of that year. Mr. Thompson was prevented by want of health from performing the task assigned him by the Society. We do not know whether the subject of Dr. Rush's Discourse grew out of a knowledge of Mr. Thompson having intended to discuss the subject of the Indians, or of any suggestion offered by this gentleman, who had been adopted by these people into one of their tribes, and was designated by them under a name signifying "the man of truth." In the fermentation of men's minds at the expectation of the coming contest between the colonies and the mother country, in which Dr. Rush was destined to take a conspicuous share, it must be a matter of some surprise that he could apply himself actively to any investigation not actually forced upon him in his professional walks, and his professorial duties in the College of Philadelphia. He was at the time only twenty-nine years of age.

After announcing the subject of his discourse, Dr. Rush points out the difficulties of an inquiry of this nature, and asks: "How shall we distinguish between the original diseases of the Indians, and those contracted from their intercourse with Europeans? By what arts shall we persuade them to receive their remedies? And lastly, how shall we come at the knowledge, facts, or cloud of errors in which the credulity of the Europeans and the superstition of the Indians have involved both their diseases and their remedies?" "In the earliest accounts we have of them we find them cultivating a spot of ground. The maize is an original grain among them. The different dishes of it which are in use among the white people still retain Indian names." Dr. Rush next inquires into the customs among these people which are known to influence diseases. Accordingly, he proceeds to speak of the birth and treatment of their children, their diet, the customs which are peculiar among the sexes, and finally those which are common to them both.

Under the first of these heads, the author very truly remarks, that much of the future health of the body depends upon its original stamina. A child born of healthy parents always brings into the world a system formed by nature to resist the causes of diseases. The treatment of children among the Indians tends to secure their hereditary firmness of constitution. Their first food is their mothers' milk. To harden them against the action of heat and cold (the natural enemies of life among the Indians) they are plunged into cold water. In order to facilitate their being moved from place to place, and at the same time to preserve their shape, they are tied to a board, where they lie on their backs for six, ten, or eighteen months. This forced inaction of the spinal muscles and those of the back generally cannot be called a natural or a healthy posture.

"A child generally sucks its mother till it is two years old, and sometimes longer. It is easy to conceive how much vigor their bodies must acquire from their simple but wholesome nourishment. The appetite we sometimes observe in children for flesh is altogether artificial. The peculiar irritability of the system in infancy forbids stimulating aliment of all kinds. Nature never calls for animal food till she has provided the child with those teeth which are necessary to divide it. I shall not undertake to determine how far the wholesome qualities of the mother's milk is increased by her refusing the embraces of her husband during the time of her giving suck."

In speaking of the diet of the Indians, which is of a mixed nature—animal and vegetable—the author observes, in reference to the condimental use of salt: "Although the interior parts of our continent abound with salt springs, yet I cannot find that the Indians used salt in their diet till they were instructed to do so by the Europeans." They preserve their meat from putrefaction by cutting it into small pieces, and exposing it to the sun, and in winter to the frost. "The Indians have no set time for eating, but obey the gentle appetites of nature as often as they are called by them. After whole days spent in the chase or in war, they often commit those excesses in eating to which long abstinence cannot fail of prompting them. It is common to see them spend three or four hours in satisfying their hunger. This is occasioned not more by the quantity they eat than by the pains they take in masticating it. They carefully avoid drinking water on their marches, from an opinion that it lessens their ability to bear fatigue." In another part of the discourse we read: "It is a practice among the Indians never to drink before dinner when they work or travel. Experience teaches that filling the stomach with cold water in the forenoon weakens the appetite and makes the system more sensible of heat and fatigue."

We cannot follow Dr. Rush in noting the several topics on which he discourses in succession, relating to Indian life, as it is not our purpose to give analyses of the several works which will come before us, but rather some ideas of their scope and general purposes. Subsequent and more intimate intercourse with the Indians, on the part of travelers and explorers, than that enjoyed by the author of the discourse, has modified some of the opinions held respecting the character and usages of these people. Much of the interest and value of the subject as handled by him grow out of the reflections which accompany his statement of facts, and the comparisons between civilized and savage life. In this respect the inquiry of Dr. Rush into the natural history of medicine among the Indians of North America will always retain a certain degree of freshness and insure a ready and attentive perusal.

The author goes on to speak of the late period of the appearance of the menses in Indian women, or not before they are eighteen or twenty years of age; then of their easy labors, and of there hardly being a period in the interval between the eruption and the ceasing of the menses in which they are not pregnant or giving suck. The custom of what one would call late marriages among the Indians—men seldom marry before they are thirty years of age—is assigned as a cause of considerable vigor enjoyed by them, and of entailing on their children more certain health. Tacitus is referred to as describing the same custom among the Germans, and with the like results. The customs common to both sexes are painting and the use of the cold bath. Their pigment of bear's grease mixed with clay "serves to lessen the sensibility of the extremities of the nerves; it moreover fortifies them against the action of those exhalations, which we shall mention hereafter as a considerable source of their diseases." The corroborant effects of the cold bath are also pointed out. The Indians are represented to be exempt from most of those passions which disorder the body. We do not see, however, in what manner "the turbulent effects of anger are concealed in deep and lasting resentments." The prolonged operation of revenge on the system must certainly be a powerful disturbing cause of both mental and bodily tranquillity.

It is remarkable that there are no deformed Indians. Some have suspected, from this circumstance, that they put their deformed children to death; but nature here acts the part of an unnatural mother. The severity of the Indian manners destroys them. In a note, the author qualifies his first assertion of the freedom of these people from deformity, by saying, that since the intercourse with whites some of the Indians have become deformed in their limbs. "This deformity, upon inquiry, appears to be produced by those accidents, quarrels, etc., which have been introduced among them by spirituous liquors." In reference to their diseases, it is broadly affirmed, that fevers constitute the only diseases among the Indians, with a specification, that the first division is into those "of the inflammatory kind," produced by cold and heat, such as pleurisies, peripneumonies, and rheumatisms. "Those which are produced by the insensible qualities of the air, or by putrid exhalations, are intermitting, remitting, inflammatory, and malignant, according as the exhalations are combined with more or less heat or cold. The dysentery (which is an Indian disease) comes under the class of fevers. It appears to be the *febris introversa* of Sydenham." The small-pox and the venereal disease were communicated to the Indians of North America. "Nor can I find," says Dr. Rush, "that they were ever subject to the scurvy." The remark by the author, that he had only heard of two or three cases of gout among the Indians, and then only among those who had learned the use of rum from the white people, is followed by an inquiry into the reasons why the gout does not appear more frequently among that class of civilized people who consume the greatest quantity of rum? The explanations offered are of a purely speculative nature, and instead of an inquiry into the comparative effects, physiological and pathological, of fermented and distilled liquors, the author solves the difficulty by such language as the following: "The effects of wine, like tyranny in a well-formed government, are felt first in the extremities; while spirits, like a bold invader, seize at once upon the vitals of the constitution." This over-readiness, not merely to illustrate by remote analogies, but to adduce them as actual steps in the causation, was one of the infirmities in Dr. Rush's mode of argument. But few instances of insanity were found among the Indians, and these were of "melancholy" and "madness; "but none of "fatuity." The author could not find any accounts of diseases from worms among them—an exemption which has not been corroborated by later observers. Dentition is said to be unaccompanied by disease in their children. Dr. Rush concludes that the only natural outlets of human life with the Indians are fevers, old age, casualties, and war. This last is declared by the writer to be "nothing but a disease; it is founded on the imperfections of political bodies, just as fevers are founded on the weakness of the animal body."

The remedies of the Indians, "like their diseases, are simple and few in number." Fevers are fancifully represented by Dr. Rush to be the principal instruments in the hands of nature to remove the evils which threaten dissolution. He divides the remedies used by the Indians into natural and artificial. His remarks on these are suggestive.

"To the class of natural remedies belongs the Indian practice of abstracting from their patients all kinds of stimulating aliment. The compliance of the Indians with the dictates of nature in the early stage of a disease no doubt prevents, in many cases, their being obliged to use any other remedy. They follow nature still closer in allowing their patients to drink plentifully of cold water, this being the only liquor a patient calls for in a fever.

"Sweating is likewise a natural remedy. It was probably suggested by observing fevers to be terminated by it. I shall not inquire how these sweats are essential to the crisis of a fever. The Indian mode of procuring this evacuation is as follows: The patient is confined to a close tent or wigwam, over a hole in the earth, in which a red-hot stone is placed; a quantity of water is thrown upon this stone, which instantly involves the patient in a cloud of vapor and sweat; in this situation he rushes out and plunges himself into a river, from whence he retires to his bed. If the remedy has been used with success, he rises from his bed in four-and-twenty hours perfectly recovered from his indisposition. This remedy is used not only to cure fevers, but to remove that uneasiness which arises from fatigue of body."

This kind of vapor bathing is practiced among the Russians, on a larger scale.

A third natural remedy among the Indians is purging; and vomits constitute a fourth. "The ipecacuanha is one of the many roots they employ for that purpose." The artificial remedies made use of by these people are bleeding, caustics, and astringent medicines. They confine bleeding entirely to the part affected. "Sharp stones and thorns are the instruments they use in procuring a discharge of blood." The Indians employ "something like a potential caustic in obstinate pains. It consists of a piece of rotten wood, called *punk*, which they place upon the part affected, and afterwards set it on fire; the fire gradually consumes the wood, and its ashes burn a hole in the flesh." Here we find, with but slight modification of material, the Chinese method of cauterizing with moxa. Dr. Rush was unable to say whether the Indians relied upon astringent or other vegetables for the cure of intermitting fever.

"This is a short account of the remedies of the Indians. If they are simple, they are like their eloquence, full of strength; if they are few in number, they are accommodated, as their languages are to their ideas, to the whole of their diseases."

Dr. Rush inquires next into the diseases and remedies of civilized nations. Taking Great Britain as a representative of them, he traces the origin of the diseases of her people through their customs, in the same manner as he did those of the Indians. He shows himself to be master of his subject, large and comprehensive as it is, by his exhibition of its prominent points, and appropriate remarks on each of them. He sketches rapidly, but with a firm touch, the chief causes of disease in an abuse of the non-naturals among children, added to premature studies and an original defect of stamina inherited from their ancestors. "Civilization rises in its demands on the health of women." Every phrase in his specification has the force of a picture. Not less striking are his remarks on the diseases of civilization, which extend themselves "through every class and profession among men. How fatal are the effects of idleness and intemperance among the rich, and of hard labor and penury among the poor! What pallid looks are contracted by the votaries of science from hanging over the 'sickly taper!' How many diseases are entailed upon manufacturers by the materials in which they work, and the posture of their bodies!" In speaking of the effects of war, the author gives a picture of occurrences and their causes in his own day, which might be taken to represent the disasters at Walcheren and in the Crimea, in the present century.

"War, as if too slow in destroying the human species, calls in a train of diseases peculiar to civilized nations. What havoc have the corruption and monopoly of provisions, a damp soil and an unwholesome sky, made in a few days, in an army! The achievements of British valor at the Havana, in the last war, were obtained at the expense of 9000 men, 7000 of whom perished with the West India fever. Even our modern discoveries in geography, by extending the empire of commerce, have likewise extended the empire of diseases. What desolation have the East and West Indies made of British subjects! It has been found, upon a sure calculation, that only ten of a hundred Europeans live above seven years after they arrive on the island of Jamaica."

Before proceeding to speak of the remedies of civilized nations, Dr. Rush "examines into the ability of nature in curing these diseases." By nature he understands nothing but *physical necessity*, to the exclusion of everything like intelligence from her operations, which "are all performed in obedience to the same laws which govern vegetation in plants, and the intestinal motions of fossils." He also gives a history, in the form of a series of propositions, of the operations of nature in a few of the diseases of civilized nations. These are little else than the expression of known facts in the phenomena of disease, dressed in a figurative style. His bias is shown in the concluding lines of a paragraph describing the general nature and origin of the remedies of civilized nations, in which we read, "that although physicians are in speculation the servants, yet in practice they are the masters of nature. The whole of these remedies seem contrived on purpose to arouse, assist, restrain, and control her operations." A comparative view is taken of the diseases and remedies of the Indians with those of civilized nations. The great mortality from fevers, in London, is spoken of with some enforcing figures; and the remark is made that they have been supplanted by new diseases, the offspring of luxury. The following prediction by an eminent Virginia physician is worth noting here, as it has been in process of rapid fulfillment. "Our countryman, Doctor Maclurg, has ventured to foretell that the gout will be lost in a few years, in a train of hypochondriac, hysteric, and bilious diseases."

The alleged success in prognosis of the Indians is compared with that of civilized people, little to the credit of the former. Reference is made to Hippocrates, with the following remark: "I was once an idolater at his altar, nor did I turn apostate from his worship till I was taught that not a tenth part of his prognostics corresponded with modern experience or observation." Still must we read with a respectful interest, if not veneration, the books of Hippocrates on "Prognostics" and "Prorrhetics." We shall find in the opening part of the first of these a recommendation to study the countenance of a sick person, and a description of a particular expression of the features known ever since as the *facies Hippocratica*, which every physician recognizes, in his own experience, as most truthful and accurate. So also of the posture of the patient, and his sawing the air, and catching as it were at objects which in his delirium he imagines he sees before him.

An apology is offered for the instability of the theories and practice of physic, which we do not repeat. It is in consequence of this fluctuation "being so necessarily connected with the changes in the customs of civilized nations, that old and young physicians so often disagree in their opinions and practices. And it is by attending to the constant changes in these customs of civilized nations that those physicians have generally become the most eminent who have soonest emancipated themselves from the tyranny of the schools of physic, and have occasionally accommodated their principles and practice to the changes in diseases." We are told "that the want of success in a medicine is occasioned by one of the following causes: First, our ignorance of the disease; secondly, our ignorance of a suitable remedy; thirdly, a want of efficacy in the remedy." Important truths, eloquently expressed, follow, respecting the impediments to successful practice; but we are unable to reproduce them here. Reference is made to the sinister effects on the body politic of the disease of a single prominent individual. "Justice has stopped its current, victories have been lost, wars have been prolonged and embassies delayed by the principal actors in these departments of government being suddenly laid up with a fit of the gout."

"Do the blessings of civilization compensate for the sacrifices we make of natural health, as well as of natural liberty?" In a more advanced part of the reply to this question the author justly remarks, that the complete enjoyment of health is as compatible with civilization as the enjoyment of civil liberty. Reference is made to hospitals, as "at the same time monuments of the charity and depravity of a people"—and sorrow expressed at the number of patients in the "hospital," and incurables in the "alms-house," of Philadelphia, showing that "we are treading in the enervated steps of our fellow-subjects in Great Britain." Were Dr. Rush now living, what would he say of the pauper palace, with its large population, on the other side of the Schuylkill River? "Our bills of mortality," continues the author, "likewise show the encroachments of British diseases among us. The nervous fever has become so familiar among us, that we look upon it as a national disease. Dr. Sydenham, so faithful in his history of fevers, takes no notice of it. Dr. Cadwallader informed me that it made its first appearance in this city about five-and-twenty years ago, (1750.)" Is not this fever the typhoid of the present day, the typhus mitior or slow nervous fever of English writers of the last century? It would seem from the following language held by Dr. Rush, as if phthisis were a new disease in the Colony of Pennsylvania. "It will be impossible to name the consumption without recalling to our minds the memory of some friend or relation who has perished within these few years by that disease. Its rapid progress among us has been unjustly attributed to the growing resemblance of our climate to that of Great Britain." Luxury, the attendant on civilization, would seem to have brought on its associated diseases, as we glean from the following: "The hysteric and hypochondriac diseases, once peculiar to the chambers of the great, are now to be found in our kitchens and workshops. All these diseases have been produced by our having deserted the simple diet and manners of our ancestors." A little farther on in the discourse, and the young philosopher avows himself still more decidedly to be *laudator temporis acti*, as when he says :—

"Some of you may remember the time, and our fathers have told those of us who do not, when the diseases of Pennsylvania were as few and as simple as those of the Indians. The food of the inhabitants was then simple; their only drink was water; their appetites were restrained by labor; religion excluded the influence of sickening passions; private hospitality supplied the want of a public hospital; nature was their only nurse, and temperance their principal physician."

It is admitted by the author that the proportion of old people is much greater among civilized than among savage nations, and that fewer in proportion die among the former than the latter. In order that America may resist the tendency to deterioration and decay, and retrace her steps in luxury and effeminacy, Dr. Rush gives the following advice :—

"I. Let our children be educated in a manner more agreeable to nature.

"II. Let the common people (who constitute the wealth and strength of our country) be preserved from the effects of ardent spirits. Had I a double portion of all that eloquence which has been employed in describing the political evils that lately threatened our country, it would be too little to set forth the numerous and complicated *physical* and *moral* evils which these liquors have introduced among us. To encounter this *hydra* requires an arm accustomed, like that of Hercules, to vanquish monsters. Sir William Temple tells us, that formerly in Spain no man could be admitted as an evidence in court who had once been convicted of drunkenness. I do not call for so severe a law in this country. Let us first try the force of severe manners. Lycurgus governed more by these than by his laws. '*Boni mores, non bonæ leges*,' according to Tacitus, were the bulwarks of virtue among the ancient Germans.

"III. I despair of being able to call the votaries of Bacchus from their bottle, and shall therefore leave them to be roused by the more eloquent twinges of the gout.

"IV. Let us be cautious what kind of manufactures we admit among us. The rickets made their appearance in the manufacturing towns of England. Dr. Fothergill informed me that he had often observed, when a pupil, that the greatest part of the chronic patients in the London Hospital were Spittal-field weavers."

* * * "Perhaps a pure air and the abstraction of spirituous liquors might render sedentary employments less unhealthy in America, even among men, than the populous towns of Great Britain."

Agriculture is declared to be "the basis of national health, riches, and populousness." What was then prophecy is now history, in regard to the future growth and prosperity of the people of North America. The anticipations based on the condition of the colonies have been more than realized in that of independent States united into a federal republic; and with Dr. Rush we have good cause to believe that, "America is the theatre where human nature will probably receive her last and principal literary, moral, and political honors." At the time (1774) in which the discourse was delivered, Pennsylvania contained nearly three hundred thousand inhabitants, and of this number nearly thirty thousand composed a city which was the third, if not the second, in commerce in the British empire. This was the growth in a period less than a century. Within a similar period, Philadelphia has reached to more than twice the then population of the colony, and to more than twenty times that of the city.

Seldom, if indeed ever, has it fallen to our lot to read a discourse or dissertation, in so small a compass, consisting of materials so rich, suggestive, and fruitful, and handled in so philosophical and benevolent a manner as this one, delivered before the American Philosophical Society. It alone would give Dr. Rush a place in American literature, both general and medical; but the satisfaction felt at its perusal is alloyed with regret that others have not imitated its eminent author in making investigations of a similar nature, grouped in the same instructive manner. An expression of our opinion on this discourse is due in a measure to our readers, as a reason, we will not say apology, for a fuller analysis of its contents than we can promise for other subjects which will come before us in this article. We did not intend to give a retrospective view, nor shall we be able, we fear, to furnish even a short and an appreciable notice of the general design and objects of each work.

The next production of Dr. Rush which we shall notice, is "An Account of the Climate of Pennsylvania and its Influence upon the Human Body." It is the first paper in the second volume of his "Inquiries." The limits and geographical features of the State are first described; then follows a topographical account of the City of Philadelphia. The climate of Pennsylvania is represented to have undergone a material change, judging from "the accounts which have been handed down to us by our ancestors." In the way of specification, we are told that "thunder and lightning are less frequent, and the cold of our winters and heat of our summers are less uniform than they were forty or fifty years ago. Nor is this all; the springs are much colder, and the autumns more temperate than formerly, insomuch that cattle are not housed as soon by one month as they were in former years. Within the last eight years there have been some exceptions to part of those observations. The winter of the year 1779–80 was uniformly and uncommonly cold." The cold in the winter of the year 1783–4 was as intense, but not as steady, as it was in the winter that has been described. It would seem from some facts on record, that the winter of 1739–40 was still colder than that of 1779–80.

"There are seldom more than twenty or thirty days, in summer or winter in Pennsylvania, in which the mercury rises above 80° in the former, or falls below 30° in the latter season."* The warmest weather is *generally* in the month of July. The transitions of temperature are often great and sudden. "Vegetation has been observed in all the winter months. Garlic was tasted in butter in February, 1779, and I well remember, when a school-boy, to have seen an apple orchard in full bloom and small apples on many of the trees in the month of December." "The greatest degree of heat on record in Philadelphia is 95°. The standard temperature of the air in the City of Philadelphia is 52½°, which is the temperature of our deepest wells, as also the mean heat of our common spring water." The autumn is the most agreeable season in the year in Pennsylvania. Details of the direction and character of the winds, and of the quantity of rain and snow which fell in the year, are given.

"After what has been said, the character of the climate of Pennsylvania may be summed up in a few words. There are no two successive years alike. Even the same successive seasons and months differ from each other in every year. Perhaps there is but one steady trait in the character of our climate, and that is, it is uniformly variable." Differences between the climate east of the Alleghany Mountains and that of the region west of this great dividing line are indicated.

References are made to the climate of Pekin, the capital of China, and of Madrid, the capital of Spain, both of which cities are within a few minutes of the same latitude as Philadelphia, to show how much climates are altered by local and relative circumstances.

"From a review of all the facts which have been mentioned, it appears that the climate of Pennsylvania is a compound of most of the climates in the world. Here we have the moisture of Britain in the spring, the heat of Africa in summer, the temperature of Italy in June, the sky of Egypt in the autumn, the cold

* The degrees recorded are in every instance those of Fahrenheit's thermometer.

and the snow of Norway and the ice of Holland in the winter, the tempests in a certain degree of the West Indies in every season, and the variable winds and weather of Great Britain in every month of the year."

Of the acute diseases of the countries mentioned, we have, in Pennsylvania, inflammations and fevers. It is alleged by the author, on the testimony of many aged persons, that pleurisies and inflammatory diseases of all kinds are less frequent than they were forty or fifty years ago. "It is a well-known fact that intermitting and bilious fevers have lessened or disappeared in proportion as the country has been cleared of its wood in many parts of the State. It is equally certain that these fevers have lessened or disappeared in proportion as the country has been cultivated. Heavy rains and freshets in the spring seldom produce fevers unless they are succeeded by unseasonably warm weather. Sudden changes from heat to cold, or cool weather, if they occur before the twentieth of August, seldom produce fevers. After that time they are generally followed by them. The same state of atmosphere, whether cold or warm, moist or dry, continued for a long time, without any material changes, is always healthy. Acute and inflammatory fevers were in vain looked for in the cold winter of 1779–80. The dry summer of 1782 and the wet summer of 1783 were likewise uncommonly healthy in the City of Philadelphia." "Diseases are often generated in one season and produced in another." "Valetudinarians always enjoy the most health, in Pennsylvania, in the summer and winter months. The spring in a particular manner is very unfavorable to them."

The account of the climate of Pennsylvania closes with some admonitions to the inhabitants to be more careful than they have been in their domestic architecture and in their clothing, as a means of protection against its frequent vicissitudes. They have suffered from not adopting customs which are used in hot and cold countries to guard against the extremes met with, respectively, in these regions. "Those inhabitants of Pennsylvania who have acquired the arts of conforming to the changes and extremes of our weather in dress, diet, and manners, escape most of those acute diseases which are occasioned by the sensible qualities of the air; and faithful inquiries and observations have proved that they attain to as great ages as the same number of people in any part of the world." Vital statistics show that, at the present day, there is evidence to justify a still more favorable statement than that made by Dr. Rush in his time.

"An Inquiry into the Influence of Physical Causes upon the Moral Faculty" was delivered before the American Philosophical Society, on the twenty-seventh day of February, 1786. Twelve years before, or in 1774, Dr. Rush had addressed this society as a subject of George III., and an inhabitant of the Colony of Pennsylvania. Now he speaks as a citizen of an independent State soon to be knit in federative union with her sister States, and as a signer of the Declaration of their Independence, which he, for the future, loses no opportunity of strengthening and rendering available for the happiness of the people, by urging, with logic and eloquence, the necessity of suitable education for elevating their moral tone and increasing their intelligence.

Dr. Rush begins his "Inquiry" by defining the moral faculty, and correcting the error of Locke, who confounds it with reason, and of Shaftsbury, who blends it with taste; and he makes some acute observations on the doctrines of the materiality and immaterality of the soul. "St. Paul and Cicero gave us the most perfect account of it that is to be found in modern or ancient authors." The analogy is pointed out between the operation of physical causes on the intellectual faculties and of these same causes on the moral faculty. Instances are related of the total absence of this latter in persons remarkable for the power of their intellectual faculties, as in the celebrated Servin whose character is drawn by Sully. On the other hand, a highly active condition of the moral faculty may show itself with an almost obliteration of intelligence. "I once knew," said the author, "a man who discerned no one mark of reason, who possessed the moral sense or sympathy in so high a degree, that he spent his whole life in acts of benevolence. Dreams derange the intellectual faculties; they alter also, in a marked degree, the moral faculty. Sometimes there is an increase of the activity of both from the same cause." Dr. Rush on this point declares: "I have more than once heard the most sublime discourses of morality in the cell of an hospital; and who has not seen instances of patients in acute disease discovering degrees of benevolence and integrity that were not natural to them in the ordinary course of their lives?" A partial lesion of the moral faculty is not unfrequent. "I knew an instance of a woman who was exemplary in her obedience to every command of the moral law, except one. She could not refrain from stealing. What made this vice the more remarkable was, that she was in easy circumstances, and was not addicted to extravagance in anything." When unable to lay her hands on other things, she would secretly fill her pockets with bread, at the table of a friend.

With a view of supplying the defects of nosological writers, Dr. Rush names the partial or weakened action of the moral faculty, *micronomia.* The total absence of this faculty he calls *anomia.* The separation of moral from intellectual insanity was first clearly and distinctly made by Dr. Rush, and in advance of Pinel, Esquirol, Marc, and Prichard, who are often referred to, as original, by those who are ignorant of the services of the illustrious American in imparting a better knowledge and treatment of this form of mental aberration. So, also, in declaring and expounding the operation of physical causes on the moral faculty, he was in advance of Cabanis, whose treatise on the physical and moral nature of man did not appear until 1803. The first six of the twelve memoirs of which this work consists had, indeed, been read in the Institute at an earlier date, or in 1796 and 1797; but this was ten years after the subject had been fully laid open in the Inquiry now under notice, or in 1786.

Dr. Rush enumerates the physical causes which act on the moral faculty; adding pertinent observations on each of them. We dare not begin the enumeration, which would tempt us to make extracts for which we have no room. By our readers this will be considered a great privation when we tell them, that in every sentence there breathes philosophy and eloquence. Let it be borne in mind by those who think it necessary to expand the enumeration of a few simple truths into a swelling volume, that this comprehensive discourse does not occupy more than thirty moderately sized octavo pages.

Of a medico-psychological character is Dr. Rush's brief "Account of the Influence of the Military and Political Events of the American Revolution upon the Human Body." He introduces to our notice a new form of insanity which he calls *anarchia*, caused by an excess of the passion for liberty, influenced by the successful issue of the war.

The short "Inquiry into the Relation of Tastes and Aliments to each other and into the Influence of this Relation upon Health and Pleasure" contains many useful facts and a few fanciful explanations. The following is suggestive: "Those chemical medicines which decompose each other are not the only substances which defeat the intention of the prescriber. Galenical medicines, by combination, I believe frequently produce effects that are of a compound and contrary nature to their original and simple qualities." Appended remarks are made on the pleasures of eating.

"An account of the State of the Body and Mind in Old Age, with Observations on its Diseases and their Remedies," contains the result of observations made by Dr. Rush during the term of five years, upon persons of both sexes, who had passed the eightieth year of their lives. He mentions the circumstances which favor the attainment of longevity, the phenomena of mind and body which attend it, its peculiar diseases, and the remedies which are most proper to remove or moderate them. The circumstances which he enumerates as most favorable to longevity are the same as those which subsequent investigations have shown to be the most efficient.

Dr. Rush's "Inquiry into the Cause of Animal Life, in three Lectures, delivered in the University of Pennsylvania," was introductory to his course on physiology, as constituting a part of the Institutes of Medicine, of which he was at the time professor. Assuming the hypothesis of life being a forced state and the result of stimuli acting on sensitive parts and organs, the professor introduces a large amount of facts pleasantly arranged and connected by plausible theory. He shows how the vigor of the human frame and longevity are best maintained.

Appertaining to physiology and hygiene is Dr. Rush's "Inquiry into the Effects of Ardent Spirits upon the Human Body and Mind, with an Account of the Means of Preventing and of the Remedies for Curing Them." This treatise bears pleasing evidence of the keen observation of its author of all that related to man's well-being, and his benevolent earnestness in abating, if he could not remove, any practice or custom which stood in the way of social progress and happiness. Had his animated remonstrances and warning voice been listened to at the time, and found a ready response and sympathizing support among his professional brethren, not to speak of the members of other professions and those in stations of political influence, what an amount of human misery and degradation, of crime in individuals and expense to the State would have been saved! Facts, arguments, persuasion and dissuasion, are all to be found in this "Inquiry," which is a fit repertory for the use of all who would write or speak in support of temperance, and in deprecation of drunkenness and its horrible concomitant and attendant evils.

Looking for subjects more purely medical, we find two papers by Dr. Rush, on "The Cause and Cure of Pulmonary Consumption." Of these it may be said that, notwithstanding the great advances made in the study of the morbid anatomy of the lungs and the formation of tubercle, and careful investigations into the effects of remedies of different climates on the disease, they contain the main conditions for combating it with any degree of success as efficiently as those now at our disposal. Dr. Rush's elementary proposition, that pulmonary consumption is induced by predisposing debility, of which tubercles are effects, is the accredited opinion of the present day, and that on which the treatment, both therapeutical and hygienic, is founded. The same holds good of his assertion that pulmonary consumption is a primary disease of the whole system—a truth forgotten and unknown to the tribe of inhalation and syringe and sponge operators. He speaks of tracheal as well as of pulmonary consumption.

In treating of the *radical remedies* for the disease, he refers to what he had said in his "Inquiry into the Diseases, etc. of the Indians," on the effects of labor and the hardships of a camp or naval life. He joins Sydenham in strongly commending the exercise of riding on horseback, to be continued on long journeys of from six to twelve months' duration. His partiality for blood-letting in the disease was extreme.

In "Observations on the Cause and Cure of Dropsies," and "An Inquiry into the Cause and Cure of the Internal Dropsy of the Brain," Dr. Rush was among the earliest to show the connection between increased secretion of serum and increased arterial action, and to deduce a means of relief in blood-letting. "Gout" is the theme of another paper, in which the subject is treated with an undue partiality for blood-letting, but also with the characteristic ingenuity and evidences of careful observation. We can merely mention, as other subjects on which he employed his pen, Cholera Infantum, Cynanche Trachealis, Scarlatina Anginosa, Tetanus, Hydrophobia, Worms, Cure of several Diseases by the Extraction of Decayed Teeth, Measles, Influenza, the Result of Observations made upon Diseases which occurred in the Military Hospitals of the United States during the Revolutionary War, and an Account of the External Use of Arsenic in the Cure of Cancers.

Excellent advice, as applicable to the members of the medical profession at the present time as it was on the day of its delivery as a lecture, February 7, 1789, is contained in the "Observations on the Duties of a Physician and the Methods of Improving Medicine, accommodated to the Present State of Society and Manners in the United States." Our practical men, whose reading and range of thought do not go beyond a dispensatory and a hand-book on the practice of physic, would feel bewildered at the very idea of following the recommendation of Dr. Rush, for physicians to study metaphysics, writers on which he names.

"An Inquiry into the Comparative State of Medicine in Philadelphia, between the years 1760 and 1766, and the year 1809," embraces interesting notices of different localities in which fevers appeared, and of the modes of living and dress of the people in the olden time. We are told that "to Dr. Thomas Bond the City of Philadelphia is indebted for the introduction of mercury into general use, in the practice of medicine. He called it emphatically 'a revolutionary remedy,' and prescribed it in all diseases which resisted the common modes of practice." Dr. Rush concludes his retrospect by a notice of the medical opinions then prevailing among the physicians of Philadelphia, and of the causes of the reduced mortality of the city so far below the former proportion.

If we should be accused of lingering too long in our notices of Dr. Rush's mixed medical productions, our answer is that they are all of a liberalizing tendency, abound in valuable information, and are calculated to elicit emulous effects in the same line from others. Besides, to nine-tenths of the profession in the United States the writings of this great man, especially those from which we have culled, are, we fear, unknown.

As the historian of Yellow Fever in Philadelphia, Dr. Rush is better known, and with more vivid remembrance, from the conspicuous part he performed in the trying years of 1793 and 1797. To him, also, are we mainly indebted for what is known of the yellow fever of 1762 in Philadelphia. The greater part of the third and fourth volumes of the "Inquiries and Observations" is taken up with a succession of histories of this disease, which he calls more generally "Bilious Yellow Fever," and sometimes, as in the epidemic of 1797, "Bilious Remitting and Intermittent Yellow Fever." Preceding these histories, their author gives, under the head of "Outlines of the Phenomena of Fever," his theory, which supposes for its cause a convulsive action of the blood-vessels, and especially the arteries. There is, we are told, but one fever, and one exciting cause of fever, and that is stimulus. In his lectures on pathology he had gone still further in asserting the unity of disease. The phenomena of fever are described, but, at the same time, hypothetical explanations are offered for their occurrence, and the reader closes his perusal of the Outlines with a feeling of surprise at the ingenuity of the author and regret at its misapplication and waste on the occasion.

By his History of the Yellow Fever of 1793 and his work on Diseases of the Mind, which we cannot stop now even to analyze, Dr. Rush has acquired a wider reputation in Europe than any other American writer on medicine,—a reputation which no one can legitimately dispute with him, and which appears to be only strengthened with the progress of time.

Contemporary with Dr. Rush, and long his colleague in the University of Pennsylvania, was Dr. Benjamin Smith Barton, who is best known in the annals of medical literature by his work on the collateral science of botany. It is of an elementary cast, written on the basis of the artificial or sexual system of Linnæus, and interspersed with remarks on vegetable physiology, of a desultory nature, indicative of the habits of study of the author, which were in part the result of infirm health. This work, under the title of "Elements of Botany; or, Outlines of the Natural History of Vegetables," etc., appeared in 1803. Dr. Barton's "Contributions for an Essay toward a Materia Medica of the United States" were of an earlier date, in 1801.

The chief contributions to medical literature in Philadelphia during the closing years of the last, and the early ones of the present century, are those which involve inquiries into the origin, pathological characters, and treatment of yellow fever. Of these we may mention the treatises and memoirs of the fever of 1793, 1798, and 1799, and "Facts and Arguments in Favor of the Pestilential or Malignant Yellow Fever," by Dr. Wm. Currie. Also, Facts and Observations on the Origin, Progress, and Nature of the Fever which prevailed in certain parts of the City and Districts of Philadelphia in the summer and autumn of 1802, etc., by Drs. Currie and Cathrall. From Dr. Cathrall we have a memoir on the analysis of the black vomit. Ffirth, author of a "Treatise on Malignant Fever, with an attempt to prove its non-contagiousness," 1804, relates numerous experiments on the subject, made on animals and on his own person. Dr. Charles Caldwell took an active part in the controversial literature of yellow fever, as may be seen by his "Semi-Annual Oration on the Origin of Pestilential Diseases," delivered before the Academy of Medicine of Philadelphia, 17th December, 1798. In the Medical and Physical Memoirs of this writer there is a paper entitled "Facts and Observations Relative to the Origin and Nature of the Yellow Fever," 1801. In the same year appeared his "Address to the Philadelphia Medical Society, on the Analogies between Yellow Fever and true Plague." Of a subsequent date was an "Essay on the Pestilential or Yellow Fever as it Prevailed in Philadelphia in the year 1805," appended to his translation of Alibert on Intermittent Fevers. Of a kindred nature is Dr. Caldwell's "Anniversary Oration on the subject of Quarantine," delivered before the Philadelphia Medical Society, on the 21st January, 1807. Also, "A Reply to Dr. Haygarth's Letter to Dr. Percival on Infectious Fevers," and an "Address to the College of Physicians at Philadelphia, on the Prevention of American Pestilence," etc., 1802. Nor must we omit on this occasion the article on the subject of black vomit, in the New York Medical Repository, by Dr. Physick, who wrote but little, but that little was always to the point.

As we shall not be able, in the space yet left for us, to do more than merely enumerate the titles of the works and the names of the authors that we have yet to notice, in completion of our general sketch of the medical literature of Philadelphia, we may as well continue our references on the subject of yellow fever down to the present time, which places us in the presence of contemporary writers. After the year 1805, the city enjoyed exemption from epidemic visitation of the fever until 1820. The history of this last attack is by Dr. Samuel Jackson, Professor of the Institutes of Medicine in the University of Pennsylvania, who has given a full and interesting "Account of the Yellow or Malignant Fever, which appeared in the City of Philadelphia in the summer and autumn of 1820." This originally appeared in the first and second volumes of Dr. Chapman's Philadelphia Medical and Physical Journal. Dr. R. La Roche has written, in the Charleston Medical Journal, vol. vii., "A Statement of Facts respecting the Mortality occasioned by the Yellow Fever in the City of Philadelphia during the various epidemics from 1699 to 1820," etc.; also, "Facts and Observations on the Origin of Yellow Fever from Local Sources of Infection, as illustrated by Occurrences on Board Ship," American Journal of Medical Sciences, new series, vol. xxv.; "Observations on the Black Vomit, its Nature and Composition," etc., American Journal of Medical Sciences, xxvii.; and "Remarks on the Origin of the Yellow Fever which prevailed in Philadelphia in 1853," Transactions of the College of Physicians, new series, vol. ii. We are indebted, also, to Dr. Wilson Jewell for an account of the "Yellow or Malignant Bilious Fever in the vicinity of South Street Wharfs, Philadelphia, 1853;" likewise observations by the same writer, in Transactions of the College of Physicians. By Dr. John Hastings we have also "Lectures on Yellow Fever, its Causes, Pathology, and Treatment," 1848.

Valuable as are the several papers whose titles we have just given toward a history of the disease by Dr. La Roche, they are but specimens of what he has done in his great work on Yellow Fever, which is one of the most substantial and meritorious contributions to, we will not say merely Philadelphia, but also American medical literature.

The first links connecting Dr. Rush's productions (the last of which bears date in the year 1809) with those of the present day in Philadelphia were a "System of Anatomy for the Use of Students of Medicine," by Dr. Wistar, in 1811, and "Elements of Surgery for the Use of Students," 1808, by Dr. Dorsey. The first of these, in its dullness of style and description, formed a striking contrast with the animation and successful demonstrations of the author in his lecture-room, of which we spoke in a former article. Dr. Dorsey's work was chiefly recommended by its serving as a text-book to the lectures of Dr. Physick, whose opinions and practice it freely detailed: it also introduced to the American student a better knowledge than he could otherwise have gained of French surgery. As a literary composition, the materials of the "Elements" were hastily and crudely put together. What a contrast is offered between this (the first American work on surgery) and the full and comprehensive treatise by Dr. Gross, which has been recently published! The first giving us the doctrines of inflammation inculcated by John Hunter, and the practice of surgery, and excellent it was too, of his celebrated pupil Dr. Physick; the latter spreading before us the broad principles of the science, as resting on chemistry, histology, and pathology, and the curative indications of the art carried out by all the aids furnished by mechanical appliances of every kind, and the most fertile therapeutics.* The other works on surgery will be noticed under the general heading.

---

* Leaving for the nonce his impersonality buried in the oracular "we," the writer does not believe that he should be deprived of the privilege of alluding, in favorable terms, to a work because it happens to be by an editor of this journal. The less objection ought there to be to the freedom now claimed, when its exercise is the echo of the critical opinions of competent judges in another hemisphere.

Before naming the productions in the several departments of scientific and practical medicine *seriatim*, we would observe that there is considerable inequality in their relative extent and richness. The genius of Hare suggested some novelties in chemistry, but on this branch Philadelphia cannot lay any claim to precedence over her neighbors. Nor has physiology been progressive in this city, although ingenious experiments have been made by Drs. Joseph Klapp and Rousseau on cutaneous absorption, and by Drs. Harlan, B. H. Coates, and Lawrence on venous and lymphatic absorption. In the same line ought to be placed the inaugural thesis of Dr. Robert E. Rogers, now professor of chemistry in the medical department of the University of Pennsylvania, and the papers by Dr. J. K. Mitchell on the Penetrativeness of Fluids, originally published in the *Am. Jour. Med. Sciences.* The most original treatise, having a collateral bearing on physiology, is the "Philosophy of the Human Voice," by Dr. James Rush.

But although Philadelphia cannot offer any work on physiology with the same type of originality as the treatises of Draper and Dalton, she can point to investigations amounting to discoveries in histogeny of a basic character, on which our knowledge of function must ultimately rest, made at various times by Dr. Leidy, Professor of Anatomy in the University of Pennsylvania. We have not the time to search out, in the different journals, his valuable contributions in this line, which have already won him a truly European reputation, as our French friends term it. Of a similar tendency are his numerous contributions to comparative anatomy and the study of fossil remains or palæontology, in the Transactions of the Academy of Natural Sciences of Philadelphia, and the publications of the Smithsonian Institute.

In some departments of medical literature, the productions of Philadelphia authors are of such a stamp as give them a weight and predominance, which are admitted without dispute in other quarters. Of these we may specify in medical lexicography, Dr. Dunglison's Dictionary; in Materia Medica and Pharmacy combined, the Dispensatory of the United States, by Drs. Wood and Bache; in Medical and Dietetical Hydrology, the Treatise on Baths, etc., by Dr. Bell; and in Ethnology, the unique works of Dr. Morton on the *Crania Americana*, and *Egyptian Ethnography.* High in rank also are the treatises on Midwifery by Drs. Dewees and Charles D. Meigs. Distinctive notice has been already taken of Dr. La Roche's Treatise on Yellow Fever.

The original works of Philadelphia authors are the following:—

*Anatomy.*—Wistar, (with additions by Pancoast,) Horner; same, United States Dissector; Morton, Illustrated Anatomy; Smith and Horner, Anatomical Atlas; Allen, Practical Anatomy; Neill, Outlines of the Arteries, Nerves, and Veins, with plates; Goddard, the Nerves and Arteries, with plates; Bryant, Anatomical Examinations; Darrach, Anatomical Drawings of the Groin; Agnew, Practical Anatomy; Godman, Anatomical Investigations; Gross on the Bones, etc.

*Physiology.*—Dunglison, Human; Reese, Analysis; Rush, (James,) Philosophy of the Human Voice; Morton, Crania Americana; Caldwell, The Variety, Complexion, and Figure of the Human Species.

*Hygiene.*—Dunglison, Human Health; Bell, Regimen and Longevity; Health and Beauty; Condie, Catechism of Health; Journal of Health, four years edited and mainly written by Bell and Condie.

*Materia Medica and Therapeutics.*—Barton, (Benjamin Smith,) Contributions to Materia Medica of the United States; Chapman, Eberle, Dunglison, New Remedies; T. D. Mitchell; Biddle, Review of Materia Medica; Bell, Dictionary of; same, Baths and Mineral Waters; same, Baths and the Watery Regimen; same, Mineral and Thermal Springs of the United States.

*Materia Medica and Pharmacy.*—Coxe, American Dispensatory; Wood and Bache, United States Dispensatory; Carson, Synopsis; Griffith, (R. E.,) Universal Formulary; Ellis, Medical Formulary; Wood, Materia Medica and Pharmacology; Reese, American Medical Formulary; Parrish, (Edward,) Introduction to Practical Pharmacy; United States Pharmacopœia.

*Medical Botany.*—Barton, (William P. C.,) Carson, Griffith.

*Chemistry.*—Bache, Hare, Gardener, Medical; Rand, Medical; Booth and Morfit, Encyclopedia of; Murphy, a Review of, for students.

*Pathological Anatomy.*—Horner, Gross.

*Pathology and Therapeutics.*—Rush, Medical Inquiries and Observations; La Roche, Yellow Fever; same, Pneumonia and Malaria; Bell and Condie, Epidemic Cholera; same, Influenza; Gross, Diseases, Injuries, and Malformations of the Urinary Bladder, etc.; Chapman, Diseases of the Thoracic and Abdominal Viscera; same, Eruptive Fevers, Hemorrhage, etc; same, Compendium of Lectures, arranged by Benedict; Morton, Consumption; Jackson, Principles of Medicine; Rush, Diseases of the Mind; Mitchell, (J. K.,) the Cryptogamous or Fungoid Origin of Fever; Gerhard, the Chest; Horner's (G. R. B.) Naval Practice; Stillé, (Alfred,) Elements of Pathology;* Hasting's Yellow Fever; Morris, Caspar, Lectures on Scarlet Fever; Clymer, Fevers.

*Practice of Medicine: Systematic Works.*—Dewees, Eberle, Bell, Dunglison, Wood. *Clinical Medicine:* Gerhard.

*Surgery.*—Text-Books: Dorsey; Gibson. Operative: Pancoast.

---

* Dr. Stillé's recent work on "Therapeutics and Materia Medica" will engage our attention at an early day.

Minor: Smith, (H. H.) Bandaging and other Operations of Minor Surgery: Sargent; Hastings. Ophthalmic: Littell. Strangulated Hernia: Parrish, (Joseph,) McClellan, (George.) Foreign Bodies in the Air-Passages: Gross. Nature and Treatment of Wounds in the Intestines: same. Treatise on the Urinary Organs: same. Hernia: Bryan. Dental: Fitch, Goddard, and Parker.

*Midwifery.*—Dewees, Meigs, (Charles D.,) Tucker; Warrington, Obstetric Catechism.

*Diseases of Females.*—Dewees, Meigs, (Charles D.;) the same on Diseases of the Uterus; same, on Childbed Fever; Dewees, Essays.

*Diseases of Children.*—Dewees, Condie, Meigs, (Charles D.,) Meigs, (John Forsyth,) Eberle, and T. D. Mitchell.

*Medical Jurisprudence.*—Wharton and Stillé.

*Toxicology.*—Griffith, (R. E.,) Poisons; Costill, Poisons.

*Dictionaries and Compendiums.*—Dunglison, Medical Lexicon; Cyclopedia of Medicine and Surgery, (Hays, editor;) Ludlow, Manual of Examinations; Neill and Smith, (F. G.,) Analytical Compendium of the Various Branches of Medical Science.

*Climatology and Medical Topography.*—Horner, (G. R. B.,) Observations on the Mediterranean, Spain, and Portugal; the same, Brazil and Uruguay; Forrey, on the Climate of the United States.

*Domestic Medicine.*—Coates, (Reynell,) Popular Medicine; Smith, (F. G.)

Kirkbride, Hospitals for the Insane.

Wood, Introductory Lectures and Addresses.

We have a list of the works published in Philadelphia, with annotations and additions by physicians of that city, but we are obliged to withhold it for want of room. Dr. Rush set the example of this fashion of the literary introduction of English authors to American readers. In doing so he selected recognized medical classics, viz.: Sydenham, Hillary on the Climate and Diseases of Barbadoes, Cleghorn on the Diseases of Minorca, and Pringle on the Diseases of the Army—thus procuring currency for valuable works, which, without his recommendation, would not have appeared at all.

Of almost equal number and variety of subjects are Philadelphia translations from the works of continental Europe: these are chiefly from the French. Their enumeration is prevented at this time for the reason already assigned in relation to annotated works. So, *a fortiori*, must be our silence on the list of medical works in Philadelphia republished without additions. Our lists of those three several classes number respectively 81, 39, and 241. To these may be added 30 by American authors at a distance, which were published in Philadelphia, making altogether 391, or nearly 400 separate works, the greater number of which have been brought out in the last forty years.

*Periodical* publication is an important feature in the medical literature of Philadelphia. For some time New York was in advance in this particular. The *New York Medical Repository* was continued from 1796 to 1822, and in that period was the recipient of a large body of valuable matter. We have not had time to engage in very minute research; but we believe that our first medical journal in Philadelphia was begun in 1804, by Dr. B. S. Barton, under the title of the *Medical and Physical Journal.* It was continued for five years, and made three volumes. Dr. John Redman Coxe originated and edited the *Medical Museum,* in six volumes, from 1805 to 1810, and one volume, new series, 1811. The next journal was the *Eclectic Repertory and Analytical Review,* by a society of physicians, represented by Drs. Otto, Hewson, and James; continued from 1811 to 1820, in ten volumes. The *Journal of Foreign Medical Literature and Science* was edited at first by Drs. Emlen and Price, for 1821 and 1822, and afterwards one volume, by Dr. Emlen, the fourth by Dr. Godman, and three subsequently by Dr. Littell. The *Medical Recorder* ran from 1818 to 1829, under different editors, of whom the chief were Drs. Eberle, George McClellan, Remington, and Calhoun. Dr. Chapman originated the *Medical and Physical Journal* in 1820. It was continued under this title until 1827; during the latter part of the time, with the assistance, at first, of Dr. Dewees, and then of Dr. Godman, and lastly of Dr. Hays. In 1827 the title of the periodical was changed to that of the *American Journal of Medical Sciences;* and on the withdrawal of Drs. Chapman and Dewees, it was placed under the charge of Dr. Hays, who has been its constant and judicious editorial guide down to the present time. Its companion, the *Medical News,* appears monthly, and has appended to it a literary department on a small scale, containing works of a standard character. The example of a more enlarged plan of conducting a medical periodical was first given by the *North American Medical and Surgical Journal,* under the direction of an editorial committee, consisting of Drs. Hodge, Meigs, Coates, Bache, and La Roche, appointed for the purpose by the Kappa Lambda Society of Philadelphia. This journal, begun in 1826, lasted until 1832; furnishing to its readers twelve volumes of matter, which obtained for it, at the time, considerable reputation. In the last two years, or from 1830 to 1832, the names of Drs. Bell, Condie, and Wood were added to the editorial corps.

The *Medical Review and Analytical Journal,* during its brief existence of three years, from 1824 to 1826, both inclusive, was edited by

Drs. Eberle and McClellan. The next journal in the order of time is the *Philadelphia Medical Examiner*. It was first issued semi-monthly, in January, 1838, under the editorial direction of Drs. Biddle and Clymer; Dr. Gerhard was also, for awhile, associate editor, as he had been previously a contributor, by furnishing valuable material for medical clinics, the reporting of which was for some years a leading feature of the *Examiner*. It assumed a monthly shape in 184–, and for a time was edited solely by Dr. Clymer; afterwards by Dr. R. M. Huston; then by Dr. F. G. Smith; by Drs. Smith and Biddle, and eventually by Dr. Hollingsworth. In 1856 the *Medical Examiner* was united to the *Louisville Medical Journal and Review*, the publication of which had been transferred to Philadelphia, and in January, 1857, the united journals were brought out as a unit under the title of the *North American Medico-Chirurgical Review*, with Drs. S. D. Gross and T. G. Richardson for its editors, with whom is now associated Dr. S. W. Gross. Last in the list is the *Medical and Surgical Reporter*, a weekly journal, under the editorial direction of Drs. S. W. Butler and R. J. Levis.

For some years Drs. Dunglison and Bell were editors of medical periodicals, which exerted a beneficial effect on the medical mind of the country. That under Dr. Dunglison's charge was the *American Intelligencer and American Medical Library*—the first part a medical journal, the second consisting of volumes selected by the editor. The *Library* was continued from 1837 to 1842; it was sent out with the *Intelligencer* by mail. The other, under the direction and supervision of Dr. Bell, was the *Eclectic Journal of Medicine*, and afterwards the *Bulletin of Medical Science*, accompanied also by a volume chosen and sometimes annotated by the editor. This was continued for upwards of ten years, or from 1836 to 1846. The circulation of so many medical works by means of the "Libraries," undoubtedly served to awaken a taste for reading and study among physicians living remote from the cities. The *Esculapian Register*, a journal of a mixed character, was published for awhile under the direction of Dr. J. R. Coxe. *The Journal of Health*, edited by Drs. Bell and Condie, had a wide circulation during the four years of its publication, from 1829 to 1833.

*The Transactions of the College of Physicians* come within the scope of our present record. The first volume appeared in 1793. A long interval was allowed to elapse from that time to 1841, when the printing of the transactions was resumed. Since then several volumes have appeared. They contain some good papers and records of conversation among the "Fellows," which are not wanting in practical value; but, as a whole, they do not give reputation to the College. Of the Transactions of the Pathological Society, the work chiefly of young men, we spoke, on a former occasion, in terms of deserved praise.

The influence of the medical schools on the medical literature of Philadelphia has been of a very decided kind, and is manifested as well in that which has been developed as in that which has not been cultivated by these institutions. The blanks left by them in medical instruction have not been filled up, or their omissions have been imperfectly supplied by the labors of authors out of the privileged corporate circle. The Institutes of Medicine, in their entireness, commanded more attention from Dr. Rush than they have since. In most of the schools they are now represented by physiology alone, to the neglect of pathology and hygiene, to say nothing of therapeutics; and hence we have no work on this entire branch, although there are not wanting treatises on its subdivisions just named. The country, for a long time, had to look for a knowledge of medical jurisprudence to the great work of the Becks; and it is only within the last few years that Philadelphia has offered an original one in that of Wharton and Stillé. In the field of general medical literature, consisting of the history of medicine and of medical schools and doctrines, with critical commentaries on the causes and effects of the successive revolutions in medical creeds and practice, Philadelphia has had no systematic or distinguished cultivators. Equal poverty is exhibited in the very attractive department of medical biography. Le Clerc, Freind, Hamilton, and Sprengel have not found either editor or publisher. The same may be said of the "Biographia Medica" of Hutchinson, and nobody among us has had the courage to attempt to continue the "Medical Bibliography" of Staff-surgeon Atkinson. We are ourselves obliged often to refer to the *Dictionnaire Historique de la Médécine* for information relating to the lives and works of eminent, and some who are not eminent, English writers. For the English translation of Reynouard's *Histoire de la Médécine*, the profession is indebted to an intelligent young physician, Dr. Comegys, of Cincinnati.

